# Supplementary material for: New alleles of D-2-hydroxyglutarate dehydrogenase enable studies of oncometabolite function in Drosophila melanogaster
Source: G3 (Bethesda). 2025 Jun 9;15(8):jkaf132. doi: 10.1093/g3journal/jkaf132 (PMC12341949; doi:10.1093/g3journal/jkaf132)
Supplement: jkaf132_Supplementary_Data [file jkaf132_supplementary_data.zip › Supplemental_Material_Legends_G3-2025-405829.docx]

**SUPPLEMENTAL FIGURES**

**Figure S1. Enrichment analysis of *D2hgdh^5-5^* mutants in comparison to controls.** MetaboAnalyst was used to perform KEGG pathway enrichment analysis of metabolites that were significantly altered in *w^1118^* *D2hgdh^5-5^* mutants males as compared with *w^1118^* controls.

**Figure S2. Enrichment analysis of *D2hgdh^12-6^* mutants in comparison to controls.** MetaboAnalyst was used to perform KEGG pathway enrichment analysis of metabolites that were significantly altered in *w^1118^* *D2hgdh^12-6^* mutants males as compared with *w^1118^* controls.

**SUPPLEMENTAL TABLE**

**Table S1.** Metabolomic analysis of the *D2hgdh* mutants compared with *w^1118^* controls. All samples contained 20 adult males. Data are normalized to sample mass and an internal d4-succininc acid standard.
