# Supplementary figures and images for: New alleles of D-2-hydroxyglutarate dehydrogenase enable studies of oncometabolite function in Drosophila melanogaster
Source: G3 (Bethesda). 2025 Jun 9;15(8):jkaf132. doi: 10.1093/g3journal/jkaf132 (PMC12341949; doi:10.1093/g3journal/jkaf132)

# Control vs *D2hgdh*<sup>5-5</sup>

## KEGG - Enrichment Overview (Top 25)

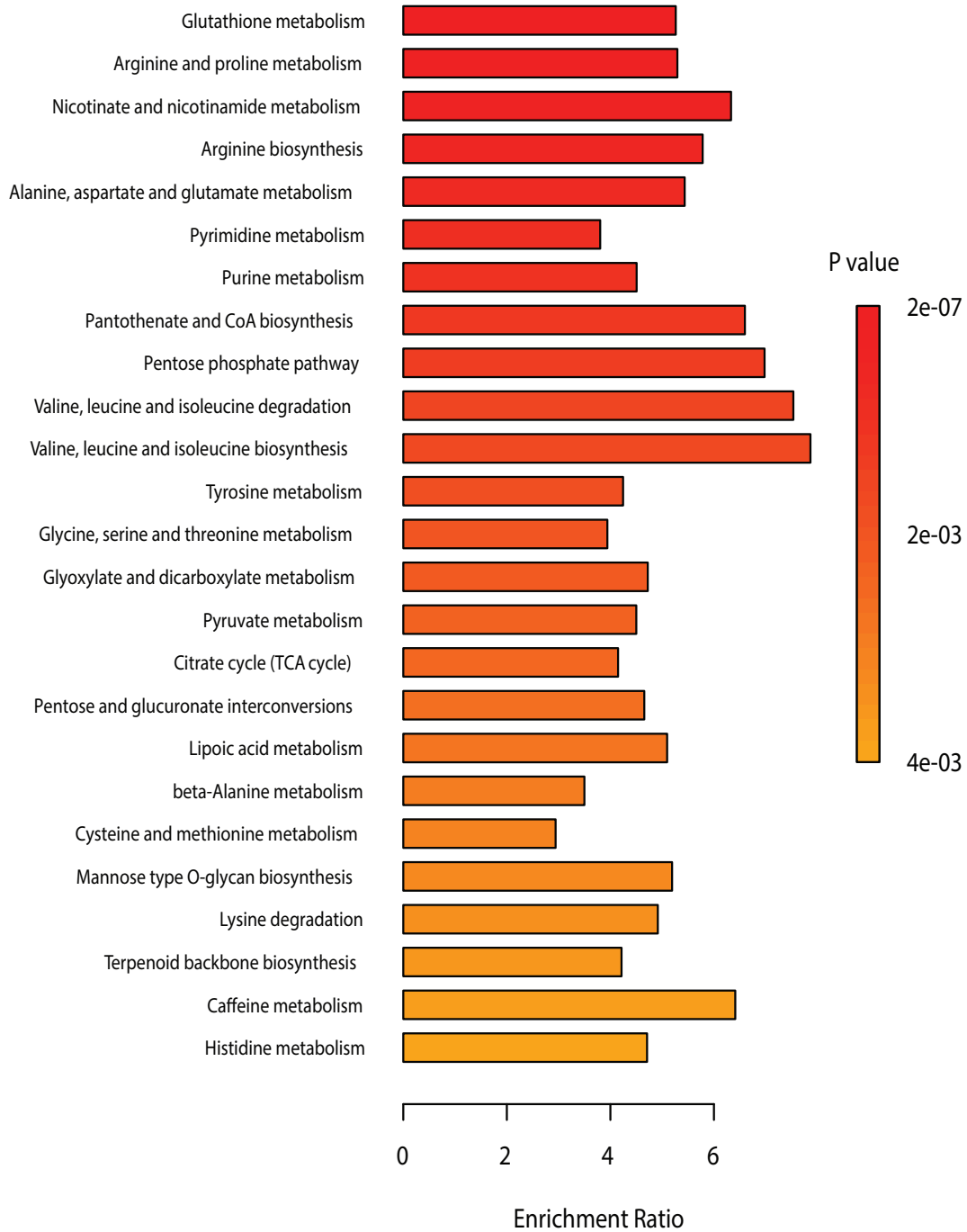

Figure S1

Supplement: jkaf132_Supplementary_Data [file jkaf132_supplementary_data.zip › Figure_S1_G3-2025-405829.pdf]

# Control vs *D2hgdh*<sup>12-6</sup>

## KEGG - Enrichment Overview (Top 25)

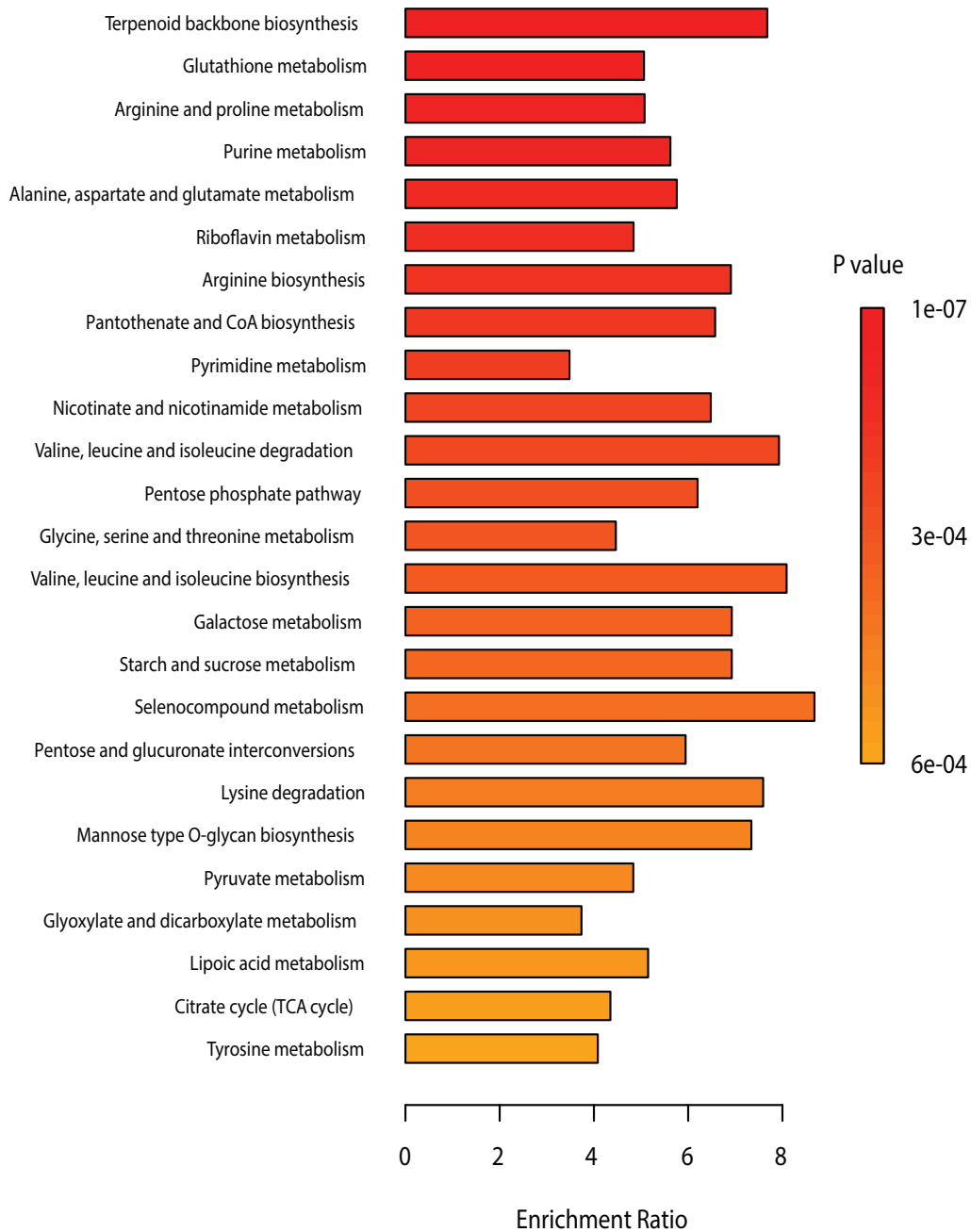

**Figure S2**

Supplement: jkaf132_Supplementary_Data [file jkaf132_supplementary_data.zip › Figure_S2_G3-2025-405829.pdf]
